# A Cross-Sectional Assessment of Complementary and Alternative Medicine (CAM) Use among Patients with Chronic Diseases (CDs) in Qassim, Saudi Arabia

**DOI:** 10.3390/healthcare10091728

**Published:** 2022-09-08

**Authors:** Maryam Farooqui, Hanan Alreshidi, Jana Alkheraiji, Suhaj Abdulsalim, Mohammed Salem Alshammari, Lamyaa Kassem, Samah Hussein, Wan Ismahanisa Ismail

**Affiliations:** 1 Department of Pharmacy Practice, Unaizah College of Pharmacy, Qassim University, Buraydah 52571, Saudi Arabia; 2 Faculty of Health Sciences, Universiti Teknologi MARA (UiTM), Campus Bertam, Kepala Batas 13200, Pulau Pinang, Malaysia

**Keywords:** complementary and alternative medicines, chronic diseases, hypertension, diabetes mellitus (DM), asthma, arthritis, chronic kidney disease (CKD), Saudi Arabia, Qassim

## Abstract

This study aims to investigate CAM use among CD patients from Qassim, Saudi Arabia and to compare CAM practice with different demographic and disease characteristics of the participants. A cross-sectional study was conducted among CD patients. During the three-months of data collection period, a total of 377 patients were approached and 208 patients participated in the study, giving a response rate of 55.17%. A *p* value of <0.05 was considered as significant. Among the study population, 94 (45.2%) patients were CAM users. Diabetes mellitus patients were the majority (48 (51.06%)) followed by hypertensive patients (34 (36.17%)). Spiritual therapies were the most common CAM followed by herbal products. Among CAM users, 41 (19.7%) patients reported disclosing CAM use to their health care providers. Among all the sociodemographic variables, gender (*p* = 0.029), marital status (*p* = 0.034) and education level (*p* = 0.047) were significantly associated with CAM use. In conclusion, the use of CAM among CD patients was relatively high in Qassim. Patients reported using CAM without disclosing to their health care providers which is a major health risk. It is critical to counsel CD patients regarding rational and informed CAM use in order to prevent harmful and unwanted effects.

## 1. Introduction

The US National Center of Complementary and Integrative Health (NCCIH) defines complementary and alternative medicine (CAM) as a non-mainstream practice that is used together with conventional medicine and “alternative” medicine as a non-mainstream practice replacing conventional medicines [1]. The integration of complementary therapies and conventional treatments known as “integrative medicine” emphasizes safety and evidence while combining the best aspects of both in a more meaningful way [2]. Although the use of CAM has associated risks, interest in CAM among healthy individuals and patients is still mounting [3]. In general, a wide range of factors have been linked to the use of CAM, including lack of basic health facilities, dissatisfaction with conventional treatment, perceived side effects of conventional medicines, positive aspects of herbal medicines, family customs and a need for religiosity or spirituality [4,5]. In addition, CAM could potentially make contributions to the effort to provide patients and healthy individuals with necessary prevention and health promotion services relating specifically to the modification of personal health behavior [6].

Chronic diseases (CDs) are reported to kill 41 million people each year, equivalent to 71% of all deaths globally. As of 2022, cardiovascular diseases account for most CD deaths, followed by cancers, respiratory diseases and diabetes [7]. Due to the prolonged nature of these disease, interest in CAM together with conventional medicines among patients with chronic diseases (CDs) is always on the rise [8,9]. In Canada, the prevalence of CAM services use was estimated to be high in patients with diabetes, asthma and epilepsy [10]. Meanwhile, in Bangladesh, CAM was an important part of the health care management plan for many patients [11]. Furthermore, CAM was used frequently to treat cancer patients in Malaysia which triggers the need for educational programs to avoid interactions with the conventional medicine (CM) for this critical disease [12]. The debilitative nature of chronic kidney disease also encouraged patients to seek other therapies than CM to delay the progression of the disease, especially those with ESRD and kidney transplantation [13]. On the other hand, a significant proportion of Jordanian patients with hypertension, diabetes and dyslipidemia tend to use CAM services along with their CM [14].

In Saudi Arabia, chronic diseases such as cardiovascular problems, diabetes, respiratory disorders and cancer are contributing significantly to mortality and morbidity [12]. Since the burden of chronic diseases is increasing, long-term management of these diseases demands costlier health care services [15]. Interest is CAM among the Saudi population for general health and for health-related issues has been well documented [16,17]. Specific to CD, only very few studies investigated the use of CAM among Saudi CD patients [18,19]. However, a comprehensive investigation of the most common types of CAM use among the Saudi chronic disease population is still lacking from Qassim, Saudi Arabia. Consequently, our study aims to describe the characteristics of patients with chronic diseases using CAM in the Qassim region. Further, we investigate the sources of recommendations, monthly expenditure and CAM disclosure to health care providers.

## 2. Materials and Methods

### 2.1. Study Design and Population

A questionnaire-based cross-sectional study was conducted among the general population of Qassim Province, Saudi Arabia from January–April 2020. The Qassim region of Saudi Arabia is densely populated with an area of 58,046 km^2^ and with an approximate population of 1,370,727 individuals [20]. Situated at the heart of the country adjacent to the geographic center of the Arabian Peninsula, Qassim is known for its agricultural land providing many traditional plants and herbs to be utilized by its population. Convenient sampling was adopted to select chronic disease patients. Initially, study participants were planned to be recruited from general hospitals of the Qassim region. However, due to COVID-19 restrictions, patients were recruited from the Qassim general population. Patients receiving their treatments from general hospitals in the Qassim region, older than 18 years, diagnosed as having chronic diseases (chronic kidney disease, hypertension, diabetes, asthma and arthritis) at any stage for the past 6 months and receiving conventional treatments were included in the study. Pregnant patients, patients with psychiatric illnesses and having chronic diseases other than the study focus were excluded from the study. Patients who declined to sign the consent form or were unable to give an interview were also excluded. Online interviews using a questionnaire were conducted with selected CD patients after informed consent was obtained.

### 2.2. Sample Size

The sample size for the survey was calculated using a 20% anticipated prevalence of CAM use, and with a 95% level of confidence and a 5% margin of error the estimated sample size was 377. The sample size was estimated using Epi Info software package version 7.2 (Centers for Disease Control and Prevention, Atlanta, GA, USA). 

### 2.3. Study Tool

A 20-item questionnaire comprising four sections was used for data collection. In addition to demographic and disease information, questions were asked about CAM, such as the types, frequency, reasons, sources of information and disclosure of CAM use to their doctors. Current and previous usage of CAM approaches (dietary and nutritional supplements, herbal products, multivitamins and supplements, aromatherapy, spiritual therapies) were evaluated. The primary version of the questionnaire was developed through an extensive literature review in the English language [12,13,16]. It was later translated into the Arabic language using standard translating procedures by native Arabic speakers [21,22]. Face and content validity of the questionnaire were measured by experts in CAM research. A cognitive debriefing was conducted with 10 CD patients (two from each selected CD). Questions ambiguous to the participants were either removed or changed. The Arabic version of the questionnaire was tested for its reliability and validity. Internal consistency was assessed by using Cronbach’s alpha (α = 0.8) and was found to be in acceptable ranges [23]. Data from piloting were not included in the final analysis.

### 2.4. Definition and Classification of CAM

For the purpose of this study, the use of CAM was defined as “any type of CAM used more than once during the six months after a diagnosis of CD”. CAM was classified according to the National Center for Complementary and Integrative Health [24,25]. Patients were specifically asked if they used CAM for general health or for the treatment of CD (included in the study), or for the management of other chronic conditions. Since five times daily prayers and recitation of Al Quran (holy book of Muslims) are daily rituals of Muslim participants, spiritual therapies were explained to them as practices other than their daily practices that were specifically performed for their chronic conditions.

### 2.5. Data Collection

The interviews were conducted by two final year pharmacy students who were trained in interviewing and questionnaire administration skills. Students were well informed and trained in the study objectives and several herbal and medicinal plants were covered in their herbal medicine, clinical nutrition, nutraceuticals and pharmacognosy courses which made them familiar with non-pharmacological, alternative and complementary therapies. Prior to the interview, patients were contacted through a phone call or WhatsApp message to participate in the study. After explaining the study objectives and obtaining consent, an interview date was fixed as per each patient’s convenience. A Google Meet link was shared a day before the interview. Patients were assured of the confidentiality of the information they revealed and that it would only be used strictly for research purposes. No financial aid or honorarium was offered to the patients and the participation was solely on a volunteer basis. Patients were given freedom to decline to give any information they were not comfortable to disclose. Each interview took approximately 10–15 min to complete. The study was approved by Qassim Ethics Committee (Approval no: 20-09-02).

### 2.6. Statistical Analysis

Statistical Package for Social Sciences (SPSS) v. 23.0 was used for data analysis. Descriptive statistics were used to illustrate respondents’ demographic characteristics. Categorical variables were measured as percentages while continuous variables were expressed as mean ± standard deviation. The Kolmogorov–Smirnov test was applied to affirm the nature of data distribution. Pearson’s chi-square test with likelihood ratio or Fisher’s exact test was used to assess the association between CAM use and sociodemographic variables. A *p* value of <0.05 was considered as significant.

## 3. Results

### 3.1. Demographic and Disease Characteristics of CD Patients and Their CAM Use

During the three-month data collection period, 377 patients were approached and 208 agreed to participate, giving a response rate of 55.17%. Participation could be improved if patients were met at the hospital or clinics but due to COVID-19 restrictions data collection was limited to only 208 patients. The majority of the patients were in the age range of 51–60 years (44 (21.2%)), followed by 21–30 years (37 (17.8%)). The majority of them were females (156 (75%)). Regarding marital status, 62 (29.8%) patients were married. The majority of the patients had a university degree (72 (34.6%)). Among the 208 participants, 94 (45.2%) reported using at least one type of CAM within the last six months of their chronic illness. Among all the sociodemographic variables, gender (*p* = 0.029), marital status (*p* = 0.034) and education level (*p* = 0.013) were significantly associated with CAM use. Demographic characteristics of the participants are shown in Table 1.

The subgroup analysis revealed that CAM use was the highest among diabetic patients (48 (51.06%)), followed by hypertensive patients (34 (36.17%)) and asthma patients (26 (27.65%)). There was a statistically significant (*p* = 0.042) association between DM and CAM use. However, there was no association between other chronic diseases and CAM use (Table 2).

Diabetic patients were the most likely to use CAM, followed by hypertensive patients. In the subgroup analysis, it was found that herbal products, multivitamins/minerals and spiritual therapies were most commonly used by the diabetic patients. As for the types of CAM used by the respondents, this study found that spiritual therapies (such as specific verses from the holy book Al Quran (Roqiah), drinking Zam Zam water (holy water in Muslim culture with reported medicinal properties) and supererogatory/Nawafil prayer (a type of optional Muslim salah) were the most common types of CAM used by all CD patients, followed by herbal products. Among the most common herbs reported were *Nigella sativa* (black seeds), *Trigonella foenum-graecum* (fenugreek), honey, *Curcuma longa* (turmeric). Details of types of CAM use are given in Table 3.

### 3.2. Sources of CAM Information and Monthly Expenditure

When asked about the sources of CAM used, friends and family members were the most common source of CAM use (41 (43.61%)) followed by health care providers (24 (25.53%)). The majority of the CAM users (52 (55.31%)) reported to spend less than SAR 50 (Saudi riyal) on CAM. Table 4 depicts the sources of CAM and monthly expenditures on CAM.

### 3.3. CAM Use Disclosure to Doctors

Although the CAM disclosure rate was 41 (43.6%), only 33 (35.1%) of the respondents reported that their doctors had specifically asked about CAM use. Fear of termination of treatment was among the most common reason for non-disclosure. The reasons for CAM non-disclosure are summarized in Table 5.

## 4. Discussion

The purpose of this study was to determine the pattern of CAM use among patients with CD in Qassim, Saudi Arabia. About 45.1% of the participants reported using CAM which is similar to what has been reported among Saudi patients previously [26,27]. We assessed the extent of CAM use in patients with five common chronic diseases: hypertension, diabetes mellitus, chronic kidney disease, asthma and arthritis [28,29]. Although there are studies that have reported a prevalence more than the figure reported in this study, the differences could be due to the CAM definition used, types of CAM therapies included in the study and the study sample [8,12,14]. There is a growing amount of literature on CAM use among CD patients where, due to the chronic nature of the disease, patients tend to go for alternative ways of treatment to cure side effects and to boost the immune system [8,10].

Gender was significantly associated with CAM use. Female patients reported the highest CAM use and more than half of them were unmarried, had a university degree and were employed. Additionally, there was a significantly higher proportion of CAM use in patients with the lowest income (less than SAR 5000). Similarly, in a US study, females with chronic illness were more likely to use CAM than males. Compared to individuals with less than a high school education, high school and college graduates were eighty percent and 2.12 times more likely to report CAM use [30].

Our study reported the highest CAM use among diabetic patients, which is in contrast with another study conducted in Riyadh among diabetic patients in an outpatient clinic where the prevalence of CAM practices was one quarter of the population, which is less than in our study [31]. A possible reason could be the study location where patients may not disclose their CAM use at an outpatient clinic, while in our case they were interviewed online at their convenience [32]. Further to this, a study conducted among diabetic foot patients in Jeddah reported that 21.7% used alternative topical medicines, and 31.2% used both conventional and CAM to complement each other and to avoid the tragedy of amputation [33]. So, either topical or ingestible, alone or in combination with conventional therapies, CAM use is prevalent among diabetic patients which requires proper counseling by doctors and pharmacists to avoid any interactions and unexpected side effects. A study in Riyadh at rheumatology clinics in two tertiary hospitals found that more than half of their patients were CAM users which reflects the popularity of CAM use [19]. In contrast, arthritis patients in our study reported less CAM use as compared to hypertensive and diabetic patients. Although rheumatoid arthritis patients show special interest in CAM to relieve symptoms and improve wellbeing, objective testing of CAM modalities through well-designed randomized control trials is needed to prove the safety and efficacy [34].

Spiritual therapies were the most common CAM used by our study participants which is similar to a study conducted in the southern region of Saudi Arabia [26]. A review of 73 articles on CAM use among the Saudi population revealed that spiritual therapies such as prayers and reciting the Holy Quran were the most common CAM [16]. Regardless of the disease, spiritual therapies are always the most common type of CAM reported in surveys. Patient interest in spiritual therapies could be due to ease of administration, less cost, negligible side effects and no fear of interactions with conventional therapies. Furthermore, it is anticipated that patients feel less fear disclosing their spiritual therapy use to health care providers compared to other CAMs [35,36]. Herbal therapies were the second most common CAM used by our study participants. Home remedies with natural products such as local herbs and honey have been long embedded in Arab culture. Honey is among the prophetic medicines which has been mentioned in Muslim holy scriptures and a significant amount of scientific data are available highlighting the clinical significance of honey [37].

As reported previously, the study participants frequently received suggestions and advice concerning CAM from their friends and relatives [11,13]. Despite the fact that medical professionals should be the best source of information on CAM, only a few reported consulting them for advice or conducting their own search [38]. In Saudi Arabia, it is still too early to assess how open doctors are to discussing CAM and how ready they are to answer patients’ questions about it. The CAM disclosure rate in this study is relatively better than what has been reported previously [11]. Many of the participants believe that it is not important for doctors to know about their CAM use and they have little knowledge about CAM. Lack of knowledge and training on CAM is one of the reasons doctors always feel reluctant to openly discuss CAM [39]. Moreover, perceived negative reaction from doctors, the fact that the doctor does not inquire and the idea that, since doctors work within a biomedical framework, they are unaware of CAM have been previously reported reasons for CAM non-disclosure [40]. CAM disclosure among adults with chronic conditions is critical to address and doctors must initiate CAM discussion with their patients. It raises concerns about the safety of CAM in combination with allopathic care. Future studies should focus on educating physicians about cross-cultural care as well as eliciting information about CAM use [41].

Specific to the role of pharmacists in CAM use, clinical pharmacists in wards have an important role to counsel CD patients on rational use of CAM. Since clinical pharmacists are extensively involved in pharmaceutical care planning and management, training on CAM may enhance their role in patient care. Similarly, the role of community pharmacists cannot be overlooked when it comes to the use of CAM among patients with CD. Since the Saudi population is reported to show an increased interest in vitamin and mineral preparations [42], it is important to recognize the forefront status of community pharmacist in counseling and advising patients with CD to make rational decisions on CAM. Pharmacists can guide CD patients specifically and the public in general in educating about correct CAM product selection, advising on adverse drug reactions and CAM interactions with conventional therapies. Besides that, an emphasis on CAM education including indigenous traditional medicinal plants in the pharmacy curriculum cannot be overlooked. By widening students’ views and instilling a deeper understanding of patient choices to achieve better health outcomes, the integration of CAM education into existing pharmacy courses may increase the understanding and knowledge of CAM modalities among pharmacy graduates [43].

### Limitations

There are a few limitations to this study. First, the findings of our study are restricted to patients from only one province of Saudi Arabia and hence may not be representative of all CD patients in the kingdom. Secondly, we planned to conduct face to face interviews but due to COVID-19 restrictions online surveys were conducted. This may have affected patients’ responses and disclosures about CAM use. Thirdly, the CAM concept was very new to the study participants and they were not able to recall many of the herbs they used for their health conditions. This recall bias may have led to an underreporting of the use of CAM.

## 5. Conclusions

In conclusion, individuals with CD, particularly those with diabetes and hypertension, consumed different types of CAM compared to other CD patients. Spiritual therapies were common among the study participants, highlighting their low cost, ease of administration and lack of side effects. The types of herbs used were those commonly known in Arab culture and were proven prophetic medicines. Since patients were not prompted regarding their CAM, use many of the patients decided not to disclose their CAM use to their health care providers. Therefore, it is essential for medical practitioners to be more watchful when it comes to CD patients using CAM, and they must regularly check patients for any negative consequences of CAM. If there are any clear advantages for CD patients, more research on the safety and effectiveness of CAM in the management of CD needs to be carried out.

## Figures and Tables

**Table 1:** Demographic characteristics of the CD patients and their CAM use.

Characteristics		No. (%) N = 208	CAM Use		*p* Value
			Yes n = 94	No n = 114	
**Age**	16–20 years	28 (13.5)	7 (25.00)	21 (75.00)	
	21–30 years	37 (17.8)	18 (48.64)	19 (51.35)	
	31–40 years	36 (17.3)	17 (47.22)	19 (52.77)	
	41–50 years	35 (16.8)	17 (48.57)	18 (51.42)	
	51–60 years	44 (21.2)	21 (47.72)	23 (52.27)	
	>60 years	28 (13.5)	14 (50.00)	14 (50.00)	0.371
**Gender**	Male	52 (25)	25 (48.07)	27 (51.92)	
	Female	156 (75)	69 (44.23)	87 (55.76)	0.029 *
**Marital status**	Unmarried	117 (56.3)	62 (52.99)	55 (47.00)	
	Married	62 (29.8)	23 (37.09)	39 (62.90)	0.034 *
	Divorced	4 (1.9)	0 (0.00)	4 (100.00)	
	Widowed	25 (12)	9 (36.00)	16 (64.00)	
**Education level**	Primary	28 (13.5)	16 (57.14)	12 (42.85)	
	Middle school	21 (10.1)	5 (23.80)	16 (76.19)	
	Secondary	67 (32.2)	24 (35.82)	43 (64.17)	
	Diploma/matriculation	19 (9.1)	11 (57.89)	8 (42.10)	
	University degree	72 (34.6)	26 (36.11)	46 (63.88)	0.047 *
	Post-graduate degree	3 (1.4)	3 (100.00)	0 (0.00)	
	Never went to school	19 (9.1)	8 (42.10)	11 (57.89)	
**Employment status**	Unemployed	42 (20.2)	17 (40.47)	25 (59.52)	
	Employed	64 (30.8)	34 (53.12)	30 (46.87)	
	Retired	20 (9.6)	12 (60.00)	8 (40.00)	
	Home maker	41 (19.7)	18 (43.90)	23 (56.09)	0.712
	Student	38 (18.3)	12 (31.57)	26 (68.42)	
	Student and employed	1 (0.5)	1 (100.00)	0 (0.00)	
	Businessman	2 (1)	0 (0.00)	2 (100.00)	
**Monthly income**	Less than SAR 5000	119 (57.2)	55 (46.21)	64 (53.78)	0.911
	SAR 5000–10,000	58 (27.9)	26 (44.82)	32 (55.17)	
	More than SAR 10,000	31 (14.9)	13 (41.93)	18 (58.06)	

* Statistically significant. *p* value was calculated using chi-square test. Abbreviations: CAM, complementary and alternative medicine; CD, chronic disease.

**Table 2:** Disease characteristics of CD patients and CAM use.

Disease Characteristics		No. (%) N = 208	CAM Use		*p* Value
			Yes n = 94	No n = 114	
**Hypertension**	Yes	68 (32.7)	34 (50.00)	34 (50.00)	
	No	140 (67.3)	60 (42.85)	80 (57.14)	0.332
**Diabetes mellitus**	Yes	92 (44.2)	48 (52.17)	44 (47.82)	0.042 *
	No	116 (55.8)	46 (39.65)	70 (60.34)	
**Asthma**	Yes	58 (27.9)	26 (44.82)	32 (55.17)	0.948
	No	150 (72.1)	68 (45.33)	82 (54.66)	
**Arthritis**	Yes	44 (21.2)	23 (52.27)	21 (47.72)	0.288
	No	164 (78.8)	71 (43.29)	93 (56.70)	
**CKD**	Yes	5 (2.4)	2 (40.00)	3 (60.00)	
	No	203 (97.6)	92 (45.32)	111 (54.67)	0.813

* Statistically significant. *p* value was calculated using chi-square test. Abbreviations: CAM, complementary and alternative medicine; CD, chronic disease; CKD, chronic kidney disease.

**Table 3:** Types of CAM used by CD patients.

Type of CAM Therapies	Chronic Diseases				
	Hypertension No. (%)	Diabetes No. (%)	Asthma No. (%)	Arthritis No. (%)	CKD No. (%)
Dietary supplement	7 (7.44)	7 (7.44)	3 (3.19)	8 (8.51)	0 (0.00)
Herbal product	15 (15.95)	22 (23.40)	14 (14.89)	11 (11.70)	1 (1.06)
Multivitamins and Supplements	13 (13.82)	22 (23.40)	9 (9.57)	14 (14.89)	1 (1.06)
Spiritual therapies	25 (26.59)	35 (37.23)	11 (11.70)	17 (18.08)	1 (1.06)
Aromatherapy	1 (1.06)	1 (1.06)	3 (3.19)	1 (1.06)	0 (0.00)

Total percentage may not be 100% due to the choice given for multiple responses.

**Table 4:** Sources of CAM information and monthly expenditure on CAM.

Variables	Frequency (%) N = 94
**Sources of recommendation**	
Own search	22 (23.40)
Recommended by family members or friends	41 (43.61)
Recommended by physicians/pharmacists/nurses	24 (25.53)
Recommended by other chronic disease patients	13 (13.82)
Social media and internet/newspaper	18 (19.14)
**Monthly expenditure on CAM in SAR**	
<50	52 (55.31)
51–100	25 (26.59)
101–200	9 (9.57)
201–300	1 (1.06)
301–400	2 (2.12)
>400	5 (5.31)

Total percentage may not be 100% due to the choice given for multiple responses. SAR: Saudi riyal = USD 0.27.

**Table 5:** Participants CAM use disclosure and reasons for non-disclosure to doctors.

Variables		Frequency (%) N = 94
Did you disclose your CAM use to your doctor/health care provider?	Yes	41 (43.6)
	No	53 (56.3)
Why did you not mention it to your doctor? N = 53		
I thought it is not important for my doctors to know about CAM	27 (50.9)	
I thought my doctor has little or no knowledge about CAM	21 (39.6)	
I thought the doctor would disagree	10 (4.8)	
I thought my doctor will stop my treatment	35 (16.8)	
They never ask I never tell	32 (15.4)	
The doctor already prescribes CAM	10 (4.8)	

Total percentage may not be 100% due to the choice given for multiple responses.

## Data Availability

Data supporting the findings of this study are available from the corresponding author on request. The information is not available to the public due to privacy or legal constraints.

## References

[1] National Center for Complementary and Integrative Health (NCCIH) Complementary, Alternative, or Integrative Health: What’s in a Name? Complementary, Alternative, or Integrative Health: What’s In a Name?|NCCIH. nih.gov.

[2] Bodeker G., Ong C.K. (2005). WHO Global Atlas of Traditional, Complementary and Alternative Medicine (Vol. 1). World Health Organization. https://www.who.int/publications/i/item/9241562862.

[3] El-Dahshan N.A., Ismail M.A., Metwally A.S. (2015). Use of complementary and alternative medicine among families having patients with chronic diseases: El-mahsama village-ısmailia governorate. Therapy.

[4] Farooqui M., Hassali M.A., Shatar A.K.A., Shafie A.A., Seang T.B., Farooqui M.A. (2012). Complementary and alternative medicine (CAM) use by Malaysian oncology patients. Complementary Ther. Clin. Pract..

[5] Singh A., Dixit S. (2021). A study on the motivation of Indian patients to consult complementary and alternative medicine practitioners to treat coronary artery disease. Int. J. Health Plan. Manag..

[6] Davis M.A., West A.N., Weeks W.B., Sirovich B.E. (2011). Health behaviors and utilization among users of complementary and alternative medicine for treatment versus health promotion. Health Serv. Res..

[7] Noncommunicable Diseases. https://www.who.int/news-room/fact-sheets/detail/noncommunicable-diseases.

[8] Hasan S.S., Ahmed S.I., Bukhari N.I., Loon W.C.W. (2009). Use of complementary and alternative medicine among patients with chronic diseases at outpatient clinics. Complementary Ther. Clin. Pract..

[9] Eckert M., Amarell C., Anheyer D., Cramer H., Dobos G. (2018). Integrative pediatrics: Successful implementation of integrative medicine in a german hospital setting—concept and realization. Children.

[10] Metcalfe A., Williams J., McChesney J., Patten S.B., Jetté N. (2010). Use of complementary and alternative medicine by those with a chronic disease and the general population-results of a national population based survey. BMC Complementary Altern. Med..

[11] Shahjalal M., Chakma S.K., Ahmed T., Yasmin I., Mahumud R.A., Hossain A. (2022). Prevalence and determinants of using complementary and alternative medicine for the treatment of chronic illnesses: A multicenter study in Bangladesh. PLoS ONE.

[12] Farooqui M., Hassali M.A., Shatar A.K.A., Farooqui M.A., Saleem F., ul Haq N., Othman C.N. (2016). Use of complementary and alternative medicines among Malaysian cancer patients: A descriptive study. J. Tradit. Complementary Med..

[13] Shamsuddin N., Karuppannan M., Hafiz Wan Md Adnan W.A., Farooqui M., Gnanasan S. (2019). Pattern of complementary and alternative medicine (CAM) use among patients with chronic kidney disease. Complement. Ther. Clin. Pract..

[14] Wazaify M., Alawwa I., Yasein N., Al-Saleh A., Afifi F.U. (2013). Complementary and alternative medicine (CAM) use among Jordanian patients with chronic diseases. Complementary Ther. Clin. Pract..

[15] Alqunaibet A., Herbst C.H., El Saharty S., Algwaizini A. (2021). Noncommunicable Diseases in Saudi Arabia: Toward Effective Interventions for Prevention. International Development in Focus.

[16] Alrowais N.A., Alyousefi N.A. (2017). The prevalence extent of Complementary and Alternative Medicine (CAM) use among Saudis. Saudi Pharm. J..

[17] Elolemy A.T., AlBedah A.M. (2012). Public knowledge, attitude and practice of complementary and alternative medicine in Riyadh region, Saudi Arabia. Oman Med. J..

[18] Alanizy L., Almatham K., Al Basheer A., Al Fayyad I. (2020). Complementary and alternative medicine practice among Saudi patients with chronic kidney disease: A cross-sectional study. Int. J. Nephrol. Renovasc. Dis..

[19] Almuhareb A.M., Alhawassi T.M., Alghamdi A.A., Omair M.A., Alarfaj H., Alarfaj A., Alomari B.A., Alblowi M.S., Almalag H.M. (2019). Prevalence of complementary and alternative medicine use among rheumatoid arthritis patients in Saudi Arabia. Saudi Pharm. J..

[20] Ministry of Interior (2022). Emirate of Al-Qasim Province. www.moi.gov.sa.

[21] Behling O., Law K.S. (2000). Translating Questionnaires and Other Research Instruments: Problems and Solutions.

[22] Harkness J.A., Schoua-Glusberg A. (1998). Questionnaires in Translation. ZUMA-Nachr. Spez..

[23] Santos J.R.A. (1999). Cronbach’s alpha: A tool for assessing the reliability of scales. J. Extension.

[24] Wieland L.S., Manheimer E., Berman B.M. (2011). Development and classification of an operational definition of complementary and alternative medicine for the Cochrane collaboration. Altern. Ther. Health Med..

[25] Ng J.Y., Dhawan T., Dogadova E., Taghi-Zada Z., Vacca A., Wieland L.S., Moher D. (2022). Operational definition of complementary, alternative, and integrative medicine derived from a systematic search. BMC Complementary Med. Ther..

[26] Alghamdi M., Mohammed A.A., Alfahaid F., Albshabshe A. (2018). Herbal medicine use by Saudi patients with chronic diseases: A cross-sectional study (experience from Southern Region of Saudi Arabia). J. Health Spec..

[27] Albassam A.A., Alanazi A., Alhaqbani N., Alhoti F., Almalki Z.S., Alshehri A.M., Alzahrani J. (2021). The potential of drug-herbal interaction among patients with chronic diseases in Saudi Arabia. Complementary Ther. Clin. Pract..

[28] Almezaal E.A.M., Elsayed E.A.H., Javed N.B., Chandramohan S., Mohammed A.M. (2021). Chronic disease patients’ satisfaction with primary health-care services provided by the second health cluster in Riyadh, Saudi Arabia. Saudi J. Health Sci..

[29] Mokdad A. (2016). Saudi Health Interview Survey Finds High Rates of Chronic Diseases in the Kingdom of Saudi Arabia. https://www.healthdata.org/news-release/saudi-health-interview-survey-finds-high-rates-chronic-diseases-kingdom-saudi-arabia#:~:text=RIYADH%20%E2%80%93%20Obesity%2C%20diabetes%2C%20high,at%20the%20University%20of%20Washington..

[30] Mbizo J., Okafor A., Sutton M.A., Leyva B., Stone L.M., Olaku O. (2018). Complementary and alternative medicine use among persons with multiple chronic conditions: Results from the 2012 National Health Interview Survey. BMC Complementary Altern. Med..

[31] Al-Eidi S., Tayel S., Al-Slail F., Qureshi N.A., Sohaibani I., Khalil M., Al-Bedah A.M. (2016). Knowledge, attitude and practice of patients with type 2 diabetes mellitus towards complementary and alternative medicine. J. Integr. Med..

[32] Foley H., Steel A., Cramer H., Wardle J., Adams J. (2019). Disclosure of complementary medicine use to medical providers: A systematic review and meta-analysis. Sci. Rep..

[33] Bakhotmah B.A., Alzahrani H.A. (2010). Self-reported use of complementary and alternative medicine (CAM) products in topical treatment of diabetic foot disorders by diabetic patients in Jeddah, Western Saudi Arabia. BMC Res. Notes.

[34] Moudgil K.D., Berman B.M. (2014). Traditional Chinese medicine: Potential for clinical treatment of rheumatoid arthritis. Expert Rev. Clin. Immunol..

[35] Farooqui M., Hassali M.A., Shatar A.K.A., Shafie A.A., Farooqui M.A., Saleem F., Aljadhey H. (2012). Complementary and alternative medicines (CAM) disclosure to the health care providers: A qualitative insight from Malaysian cancer patients. Complementary Ther. Clin. Pract..

[36] Ismail W.I., Hassali M.A.A., Farooqui M., Saleem F., Roslan M.N.F. (2018). Complementary and alternative medicine (CAM) disclosure to health care providers: A qualitative insight from Malaysian thalassemia patients. Complementary Ther. Clin. Pract..

[37] Sheikh B.Y. (2016). The role of prophetic medicine in the management of diabetes mellitus: A review of literature. J. Taibah Univ. Med. Sci..

[38] Verhoef M.J., Trojan L., Armitage G.D., Carlson L., Hilsden R.J. (2009). Complementary therapies for cancer patients: Assessing information use and needs. Chronic. Dis. Can..

[39] Farooqui M., Othman C.N., Hassali A.A., Saleem F., Haq N.U., Sadeeqa S. (2014). A qualitative exploration of Malaysian doctors’ perceptions towards complementary and alternative medicines (Cam). Value Health.

[40] Robinson A., McGrail M.R. (2004). Disclosure of CAM use to medical practitioners: A review of qualitative and quantitative studies. Complementary Ther. Med..

[41] Mehta D.H., Gardiner P.M., Phillips R.S., McCarthy E.P. (2008). Herbal and dietary supplement disclosure to health care providers by individuals with chronic conditions. J. Altern. Complementary Med..

[42] Alwalan S.I., Alrasheed A.A., Aldossari K.K., Al-Zahrani J.M., Alshahrani A.M., Batais M.A., Almigbal T.H. (2022). Prevalence and characteristics of multivitamin-multimineral (MVMM) use among Saudi populations in Riyadh, Saudi Arabia: A cross-sectional study. Medicine.

[43] Tiralongo E., Wallis M. (2008). Integrating complementary and alternative medicine education into the pharmacy curriculum. Am. J. Pharm. Educ..

